# Associations between prostate cancer‐related anxiety and health‐related quality of life

**DOI:** 10.1002/cam4.3069

**Published:** 2020-04-23

**Authors:** Daniel O. Erim, Antonia V. Bennett, Bradley N. Gaynes, Ram S. Basak, Deborah Usinger, Ronald C. Chen

**Affiliations:** ^1^ HEOR Modeling and Advanced Analytics Parexel International Durham NC USA; ^2^ Department of Health Policy and Management The University of North Carolina at Chapel Hill Chapel Hill NC USA; ^3^ Lineberger Comprehensive Cancer Center School of Medicine The University of North Carolina at Chapel Hill Chapel Hill NC USA; ^4^ Department of Psychiatry School of Medicine The University of North Carolina at Chapel Hill Chapel Hill NC USA; ^5^ Department of Radiation Oncology School of Medicine The University of North Carolina at Chapel Hill Chapel Hill NC USA; ^6^ Department of Radiation Oncology School of Medicine The University of Kansas Lawrence KS USA

**Keywords:** anxiety, emotional, health, life, mental, probable depression, prostate cancer, quality, role

## Abstract

**Background:**

There are uncertainties about prostate cancer‐related anxiety's (PCRA) associations with health‐related quality of life (HRQOL) and major depression, and these could affect the quality of mental healthcare provided to prostate cancer patients. Addressing these uncertainties will provide more insight into PCRA and inform further research on the value of PCRA prevention. The goals of this study were to measure associations between PCRA and HRQOL at domain and subdomain levels, and to evaluate the association between PCRA and probable (ie, predicted major) depression.

**Method:**

We analyzed secondary cross‐sectional data from the *North Carolina Prostate Cancer Comparative Effectiveness & Survivorship Study* (NC ProCESS—a population‐based cohort of prostate cancer patients enrolled shortly after diagnosis [between January 2011 and June 2013] and followed prospectively). Patient‐reported measures of PCRA and HRQOL from 1,016 enrollees who participated in NC ProCESS’s 1‐year follow‐up survey were assessed. Outcomes of interests were a) linear correlations between contemporaneous memorial anxiety scale for prostate cancer (MAX‐PC) and Short Form 12 (SF‐12) scores, and b) measures of association between indicators of clinically significant PCRA (ie, MAX‐PC > 27) and probable depression during survey contact (ie, SF‐12 mental component score ≤43).

**Results:**

PCRA measures had notable associations with SF‐12’s *mental health* subscale (assesses low mood/nervousness [*rho* = −0.42]) and *emotional role functioning* subscale (assesses subjective productivity loss [*rho* = −0.46]). Additionally, the risk of probable depression was significantly higher in participants with clinically significant PCRA compared with those without it (weighed risk ratio = 5.3, 95% confidence interval 3.6‐7.8; *P* < .001).

**Conclusion:**

Prostate cancer patients with clinically significant PCRA should be assessed for major depression and productivity loss.

## Graphical abstract

Patients with clinically significant prostate cancer‐related anxiety should be assessed for major depression (and vice versa), as well as productivity loss.

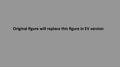

## 1 INTRODUCTION

Major depression and anxiety disorders commonly occur in cancer patients and have adverse effects on the cost and quality of cancer survivorship.^1^, ^2^, ^3^ To optimize health‐related quality of life (HRQOL), cancer care guidelines usually include evidence‐based recommendations for mental healthcare strategies (eg, regular depression screening with validated instruments).^4^, ^5^, ^6^ Prostate cancer patients stand to benefit from widespread adoption of these recommendations because (a) men are less likely than women to report mental health symptoms or seek mental healthcare,^7^, ^8^ and (b) their unmet mental healthcare needs increase over time.^9^ Prostate cancer patients are also at risk of a unique situational anxiety called Prostate Cancer Related Anxiety (PCRA).^10^, ^11^ PCRA was first described about 15 years ago and has been shown to adversely affect HRQOL.^10^, ^11^, ^12^, ^13^ Affected patients may present with anxiety symptoms, and symptoms are clinically significant (ie, require behavioral intervention) in about 10% of prostate cancer patients.^10^, ^11^, ^12^, ^13^ However, not much is known about PCRA specifically, and it may be mistaken for other anxiety disorders. ^7^, ^8^ This is important as limitations in current knowledge of PCRA may adversely affect patients’ safety. For example, PCRA has three subdomains: prostate cancer anxiety, PSA (prostate‐specific antigen) anxiety, and fear of recurrence.^10^, ^11^ However, survivorship guidelines provide care recommendations for the PSA anxiety subdomain only,^14^, ^15^ and lack of care recommendations for other PCRA subdomains may create uncertainties about the extent to which they affect patients’ HRQOL. Survivorship guidelines also state that “Survivors with significant or persistent PSA anxiety may be at heightened risk of depressive symptoms….”^14^, ^15^—however, published evidence supporting this statement is mixed and this could introduce uncertainty into clinical decision‐making on whether prostate cancer patients with clinically significant PCRA should be assessed for major depression and vice versa.^10^, ^16^, ^17^, ^18^, ^19^, ^20^ To address these uncertainties, we analyzed patient‐reported measures of PCRA and HRQOL in cross‐sectional secondary data from a study that prospectively followed a population‐based cohort of prostate cancer patients.^21^, ^22^ Our first study goal was to measure associations between PCRA and HRQOL at domain and subdomain levels using standard approaches. Our second goal was to evaluate the association between PCRA and probable depression (ie, predicted major depression [described below]) using analytic methods that reduce the risk of bias (eg, risks of PCRA and probable depression reduce over time; hence, time since prostate cancer diagnosis may bias measures of association upwards). These findings will help inform patients, clinicians, and policymakers on the potential and magnitude of significance of PCRA in prostate cancer survivors. Furthermore, if indeed PCRA is associated with depression, it will inform further research on whether early screening for and prevention of PCRA can lead to improved patient outcomes.

## 2 METHODS

### 2.1 Data

The data are from the North Carolina Prostate Cancer Comparative Effectiveness & Survivorship Study (NC ProCESS). NC ProCESS is a population‐based cohort of prostate cancer patients enrolled shortly after diagnosis (between January 2011 and June 2013) and followed prospectively; data collection continues.^21^ Potential participants were identified using North Carolina Central Cancer Registry's Rapid Case Ascertainment (RCA) system. A total of 1419 patients enrolled in the study prior to any prostate cancer treatment. Details of patient enrollment and data collection have been described elsewhere.^21^, ^22^ The current analyses included patient‐reported measures of PCRA and HRQOL from 1,016 enrollees who participated in NC ProCESS’s 1‐year follow‐up survey. IRB exemption was obtained for this analysis of de‐identified data.

### 2.2 Measures of interest

#### 2.2.1 Memorial anxiety scale for prostate cancer (MAX‐PC)

PCRA was assessed using the MAX‐PC, a validated 18‐item instrument.^10^, ^11^ MAX‐PC has three subscales for indicated PCRA subdomains: prostate cancer anxiety, prostate‐specific antigen (PSA) anxiety, and fear of recurrence. Each of the 18 items has four possible responses on a Likert scale, and all responses are scored from 0 to 3, with higher scores indicating more anxiety.^10^, ^11^ Note that MAX‐PC items 15 to 18 have responses in the reverse (ie, higher scores indicate less anxiety); these items were scored in reverse to maintain consistency with the rest of the instrument. Total MAX‐PC scores vary from 0 to 54, and patients with scores above 27 have clinically significant PCRA based on published literature.^10^, ^11^

#### 2.2.2 Short form‐12 health survey (SF‐12)

The SF‐12 (version 2) is a validated 12‐item questionnaire that measures generic HRQOL.^23^ SF‐12 is a shortened version of SF‐36, and it has 8 subscales: general health; physical functioning; physical role functioning (assesses subjective productivity loss due to physical illness); bodily pain; vitality; emotional role functioning (assesses subjective productivity loss due to mental illness); mental health (assesses low mood and nervousness); and social functioning.^23^, ^24^, ^25^, ^26^ SF‐12 item response choices are either on a Likert or binary (yes/no) scale, and responses are scored, weighed, and summed to yield physical and mental component scores that range from 0 to 100, with higher scores indicating better HRQOL. In this study, SF‐12 component and subscale scores were generated using a Stata® module developed by Niels Henrik Bruun (2016).^27^

#### 2.2.3 Others

Other measures of interest have been shown to be associated with PCRA and/or HRQOL in prior studies. These measures include Prostate Cancer Symptom Index (PCSI) scores, prostate cancer treatment type, and National Comprehensive Cancer Network (NCCN) risk group.^16^, ^18^, ^20^, ^28^, ^29^, ^30^, ^31^, ^32^, ^33^ The PCSI is a 29‐item validated instrument that assesses frequency or severity of symptoms due to prostate cancer treatments.^34^ PCSI items are grouped into four domains: urinary incontinence, urinary obstruction/irritation, bowel dysfunction, and sexual dysfunction.^34^ Responses to PCSI items are on a Likert scale, and each response is assigned a score.^34^ Item scores are summed and rescaled to vary between 0 and 100, with higher scores indicating greater dysfunction.^34^, ^35^ During NC ProCESS, the PCSI was assessed at the 12th month of follow‐up.^21^ Data on prostate cancer treatment types were abstracted from medical records and/or obtained from the cancer registry.^22^ Study participants received active surveillance, radical prostatectomy, or radiation therapy as initial treatment.^22^ NCCN risk group is the standard measure of prostate cancer aggressiveness. Prostate cancer clinical stage, Gleason score, and PSA level are used to categorize each patient's cancer as low risk, intermediate risk, or high risk. Data from study participants’ medical records were used to determine NCCN risk group.

### 2.3 Statistical analyses

#### 2.3.1 Associations between MAX‐PC and SF‐12 scores

Kolmogorov‐Smirnoff tests were used to assess for and confirm normality, while Pearson's correlation coefficients (r) were used to assess linear relationships between MAX‐PC and SF‐12 scores. Following convention, notable associations (ie, moderate or stronger correlations) exist when correlation coefficients have an absolute value of at least 0.4.^36^, ^37^

#### 2.3.2 The association between PCRA and probable depression

After reviewing evidence on associations between other types of anxiety (eg, generalized anxiety disorder) and major depression in other patient populations, we hypothesized a positive association between clinically significant PCRA and probable depression.^38^, ^39^, ^40^, ^41^ We tested this hypothesis by evaluating the relationship between indicators of clinically significant PCRA and probable depression. MAX‐PC total scores above 27 were used to create a binary indicator of clinically significant PCRA.^10^, ^11^ SF‐12 Mental Component Scores (SF‐12 MCS) of 43 or less were used to create a binary indicator of probable depression (probable because SF‐12 MCS ≤ 43 is not diagnostic).^42^ A previous study by Santos and colleagues showed this SF‐12 MCS threshold to be 73% sensitive and 90% specific for current episodes of major depression.^42^ Modified Poisson regression (ie, Poisson regression with a robust error variance) was used to measure the association between binary indicators of clinically significant PCRA (explanatory variable) and probable depression (outcome variable).^43^ Modified Poisson regression estimates risk ratios which seem easier to interpret than odds ratios.^44^, ^45^ Inverse probability weights (IPWs) were used to control confounding.^46^, ^47^ IPWs were generated with a logit regression model that had the binary indicator of clinically significant PCRA as the outcome variable, and risk factors for clinically significant PCRA (identified a priori) as right‐hand side variables (including prostate cancer symptom index scores, National Comprehensive Cancer Network [NCCN] risk categories, prostate cancer treatment type, race, age, time since prostate cancer diagnosis, and marital status).^10^, ^11^, ^16^, ^18^, ^20^, ^28^, ^29^, ^30^, ^31^, ^32^, ^33^ IPWs reduce confounding by creating a pseudo‐population in which clinically significant PCRA is independent of observed confounders. IPWs work by a) giving more analytic weights to participants with clinically significant PCRA (ie, the “exposure” group), b) giving more analytic weight to participants without clinically significant PCRA (ie, the “control” group), but who resemble those in the exposure group on observed characteristics; and c) giving less analytic weights to participants in the control group who do not resemble those in the exposure group on observed characteristics. The end result is a control group that closely resembles the exposure group in measured covariates. Weighed baseline covariates were balanced, and observations with IPWs above the 99th percentile were truncated.^46^ In sensitivity analysis, confounding was controlled by including the following patients’ characteristics as model covariates: age at cancer diagnosis, marital status, race, time since prostate cancer diagnosis, PCSI domain scores, prostate cancer treatment type, and NCCN risk category.

## 3 RESULTS

### 3.1 Characteristics of study participants

Participants’ characteristics are presented in Table 1. Among the 1,016 participants, the mean age was 65 years (range: 41‐81 years), and the cohort was sociodemographically diverse with 26% being non‐White and 31% having high school education or less—reflective of a population‐based cohort. The mean SF‐12 mental and physical component scores were 54 and 49, respectively. Additionally, about 8% of participants had clinically significant PCRA, and 11% had probable depression. Less than 10% of participants had missing observations, and list‐wise deletion was used in the regression analyses.

**TABLE 1:** Baseline characteristics of all NC‐ProCESS participants (N = 1016)

Characteristics	N (%) or Mean (SD)
Age, y (mean and SD)	64.60 (7.47)
Race^a^	
White	747 (73.67)
African American	247 (24.36)
Other	20 (1.98)
Educational attainment	
≤High School graduate	316 (31.10)
Some College	294 (28.94)
College graduate	406 (39.96)
Marital status^b^	
Never married	37 (3.64)
Married	819 (80.61)
Other	159 (15.66)
Annual income^c^	
<$40 000	351 (35.70)
$40 001‐$70 000	284 (28.89)
$70 001 $90 000	131 (13.33)
>$90,000	217 (22.08)
Employment status	
Employed	435 (42.82)
Unemployed	30 (2.95)
Retired	480 (47.24)
Disabled, not working	71 (6.99)
Health insurance status^d^	
Insured	982 (96.94)
Uninsured	31 (3.06)
NCCN risk categories	
Low risk	493 (49.20)
Intermediate risk	315 (37.43)
High risk	134 (13.37)
Prostate cancer treatment type	
Active surveillance/no treatment	280 (27.56)
Radiation therapy	378 (31.00)
Radical prostatectomy	421 (41.44)
SF‐12 mental component score (mean & SD)^e^	54.29 (8.18)
SF‐12 physical component score (mean & SD) ^e^	48.71 (9.67)

Abbreviations: MAX‐PC, memorial anxiety scale for prostate cancer; NCCN, National Comprehensive Cancer Network; PSA, prostate cancer antigen; RP, radical prostatectomy; RT, radiation therapy; SD, standard deviation.

^a^ 2 missing.

^b^ 1 missing.

^c^ 33 missing.

^d^ 3 missing.

^e^ 8 missing.

### 3.2 Associations between MAX‐PC and SF‐12 scores

A matrix of correlation coefficients between MAX‐PC and SF‐12 scores is presented in Table 2. All correlation coefficients were negative and statistically significant at an alpha of 0.05. MAX‐PC total scores had a notable association with SF‐12 mental component scores (r = −0.44) but not with SF‐12 physical component scores (r = −0.25). Notable associations were also seen in the following pairings: MAX‐PC total scores and SF‐12’s emotional role functioning subscale scores (r = −0.46); MAX‐PC total scores and SF‐12’s mental health subscale scores (r = −0.42); and MAX‐PC’s fear of recurrence subscale scores and SF‐12’s emotional role functioning subscale scores (r = −0.43). Among MAX‐PC subscales, the PSA anxiety subscale always had the least correlation coefficients with SF‐12 component and subscale scores.

**TABLE 2:** A matrix of correlation coefficients between short form 12 (SF‐12) and memorial anxiety scale for prostate cancer (MAX‐PC) scores

SF‐12 scores	Prostate cancer anxiety subscale scores	PSA anxiety subscale scores	Fear of recurrence subscale scores	MAX‐PC total scores
SF‐12 subscales scores				
Social functioning	−0.35	−0.29	−0.35	−0.39
Emotional role functioning	−0.39	−0.33	−0.43	−0.46
Mental health	−0.38	−0.27	−0.37	−0.42
General health	−0.27	−0.14	−0.34	−0.31
Physical function	−0.26	−0.18	−0.29	−0.30
Physical role functioning	−0.29	−0.14	−0.37	−0.34
Bodily pain	−0.23	−0.18	−0.27	−0.27
Vitality	−0.22	−0.12	−0.30	−0.26
SF‐12 Physical component scores	−0.22	−0.11	−0.29	−0.25
SF‐12 Mental component scores	−0.39	−0.31	−0.39	−0.44

Note

NB: All linear correlations are statistically significant at alpha 0.05. Additionally, the sample size varied between 988 and 1015 per correlation coefficient. Notable correlations had coefficients with an absolute value of at least 0.4.^36^, ^37^

Abbreviation: PSA, prostate‐specific antigen.

### 3.3 Associations between clinically significant PCRA and probable depression

The distribution of participants by the presence of clinically significant PCRA and/or probable depression is presented in Table 3. The prevalence of probable depression was up to seven times higher in participants with, compared with those without, clinically significant PCRA. Associations between probable depression and clinically significant PCRA are also presented in Table 3. We found support in our hypotheses in the weighed risk ratio (ie, 5.3; 95% confidence interval 3.6‐7.8; *P*‐value < .001) and the adjusted risk ratio from sensitivity analysis (ie, 4.5; 95% confidence interval 3.2‐6.6; *P*‐value < .001).

**TABLE 3:** The association between clinically significant PCRA and probable depression

	No probable depression	Probable depression	Unadjusted RR (n = 988)	Weighed RR (n = 936)	Adjusted RR (n = 935)
	(n = 883)	(n = 105)	(95% CI)	(95% CI)	(95% CI)
CS PCRA absent	844 (95.6%)	65 (61.9%)	1.0	1.0	1.0
CS PCRA present	39 (4.4%)	40 (38.1%)	7.1 (5.1‐9.8)***	5.3 (3.6‐7.8)***	4.5 (3.2‐6.6) ***

Note

Missing observations were handled by list‐wise deletion.

Abbreviations: CI, confidence interval; CS PCRA, clinically significant PCRA; RR, risk ratio.

*** *P*‐value < .001.

## 4 DISCUSSION

The evidence presented in this study clarifies PCRA’s association with major depression and demonstrates PCRA’s notable association with subjective productivity loss (most likely through its fear of recurrence subdomain). The evidence also suggests that PSA anxiety has the least impact on HRQOL than other PCRA subdomains. These findings have several implications on policy, clinical practice, and research.

First, standards of care for distress in prostate cancer patients should include regular assessment of PCRA’s prostate cancer anxiety and fear of recurrence subdomains.^14^ This seems reasonable as current survivorship guidelines do not provide care recommendations for these PCRA subdomains. Second, prostate cancer patients with a diagnosis of major depression should be assessed for clinically significant PCRA and vice versa. Related recommendations in survivorship guidelines also need to be clarified; specifically, the American Cancer Society prostate cancer survivorship care guidelines which state “Survivors with significant or persistent PSA anxiety may be at heightened risk of depressive symptoms” could be modified to include “Survivors with clinically significant PCRA are at heightened risk of depressive symptoms…”). Third, patients with clinically significant PCRA may need support for productivity loss. This suggestion is strengthened by the three‐way associations between major depression, productivity loss, and clinically significant PCRA.^48^ However, the link between subjective and objective productivity loss needs to be further investigated in future research.

This study has several limitations. Our strategy for identifying probable depression during survey contact (ie, SF‐12 MCS ≤ 43) is an imperfect measure of major depression (sensitivity = 73% and specificity = 90%).^42^ This imperfection may bias our measures of association toward the null. However, diagnostic tools for major depression have similar test performances (eg, Patient Health Questionnaire 9 has a sensitivity of 80% and a specificity of 92%).^49^, ^50^ Comorbidities and family history of prostate cancer were not included in the regression models due to lack of these data for analysis, and this exclusion may have introduced omitted variable bias. However, this risk may be minimal as there is a lack of evidence of an association between PCRA and indicated variables.^16^, ^28^ We used cross‐sectional data from a longitudinal study for our analyses; thus, findings only apply in the short term (when risks of major depression and PCRA are maximal). To this end, we encourage future research that assesses the robustness of our findings in the medium to long term. The data come from an observational study: hence, there is a risk of residual confounding that persists in spite of the rigorous analytic methods applied. Finally, it is pertinent to emphasize that co‐occurrence (not causation) is implied in the measure of association between clinically significant PCRA and probable depression.

The study also has several strengths. To our knowledge, this is the first study to identify a link between fear of cancer recurrence and productivity, and to demonstrate a robust association between PCRA and probable depression. Furthermore, this is a population‐based cohort composed of participants who are diverse with regards to race, education, and household income, which facilitates the generalizability of findings from this study. Lastly, the prevalence of clinically significant PCRA in this study (ie, 8%) is concordant with published estimates in the literature (ie, approximately 10%).^10^, ^11^

## 5 CONCLUSION

In a population‐based cohort of prostate cancer patients, we found evidence of PCRA’s associations with probable depression and subjective productivity loss shortly after cancer diagnosis.

## CONFLICT OF INTEREST

None.

## AUTHOR CONTRIBUTIONS

Daniel O. Erim: Conceptualization, investigation, formal analyses, writing – original draft, project management. Antonia V. Bennett: Investigation, writing – review and editing; Bradley N. Gaynes: Investigation, writing – review and editing; Ram S. Basak: Investigation, writing – review and editing; Deborah Usinger: Investigation, writing – review and editing; Ronald C. Chen: Investigation, supervision, writing – review and editing.
